# Barriers to Treatment Resulting in Multiple Coexisting Infections and Hospice in a Young Person With Acquired Immunodeficiency Syndrome (AIDS)

**DOI:** 10.7759/cureus.114177

**Published:** 2026-08-08

**Authors:** Alexandra Bartholomew, Mason Calico, John Macaulay, Catherine Loehr, Shane Sanne

**Affiliations:** 1 Department of Medicine, Louisiana State University Health Sciences Center New Orleans, New Orleans, USA

**Keywords:** antiretroviral therapy, barriers to treatment, coexisting infections, hiv aids, hospice, human immunodeficiency virus (hiv), opportunistic infections in hiv, social determinants of health, treatment non-adherence

## Abstract

Barriers to treatment in people with human immunodeficiency virus (HIV) are often multifactorial and can lead to devastating outcomes. The authors present the case of a young woman with HIV who eventually developed acquired immunodeficiency syndrome (AIDS) attributed to barriers to continuous treatment, including difficulty attending appointments, obstacles to obtaining medication, untreated depression, lack of a support system, and other unmet social needs. She unfortunately developed multiple complications related to her AIDS diagnosis, including numerous hospital admissions and several severe infections. At only 27 years old, she entered hospice.

While treatment for HIV has greatly improved, treatment interruptions continue to contribute to dangerous and potentially lethal sequelae. These barriers are often intricately intertwined with systemic failures in care delivery and support systems. This case underscores the need for more comprehensive implementation of multi-level interventions that address both individual barriers and systemic failures in the delivery of care for people with HIV. Evidence-based, patient-centered strategies that should be more widely enacted include multidisciplinary care teams with case management and social work, assistance with essential needs such as housing and food, flexible appointment scheduling, accessible medication programs, and individually tailored care delivery models. More effectively addressing social determinants of health such as housing instability, food insecurity, and transportation barriers is critical to improving care retention and treatment adherence.

## Introduction

Human immunodeficiency virus (HIV) is a retrovirus transmitted through direct exchange of bodily fluids that targets the cells of the immune system, which is essential for fighting off infections and abnormal cells. When untreated, HIV can progress to acquired immunodeficiency syndrome (AIDS), a severe condition associated with a host of infections, cancers, and other multi-organ system complications (including immune, nervous, integumentary, cardiovascular, gastrointestinal, and renal systems). Levels of CD4 cells, which are vital to the immune system, can be analyzed as a representation of the robustness of one's immunological function. A normal CD4 count is above 500 cells/mm^3^, while a count below 200 cells/mm^3^ is diagnostic for AIDS. Successful treatment of HIV can also be monitored through one's viral load, with the goal being an undetectable level indicating that the virus is unable to be transmitted. 

The first cases of HIV were reported in 1981. Between then and 2019, the estimated number of new infections in the United States rose to 2.2 million [1]. Annual incidence reached its peak in the mid-1980s; more recently, in 2019, the annual incidence of HIV in the United States fell to 34,800 [1]. Moreover, between 2018 and 2022, the United States saw a 12% decline in the estimated number of infections [2]. 

This progress can be attributed in part to both advances in treatment for those with HIV diagnoses and advances in prevention for individuals who are HIV-negative but at risk. The role of antiretroviral therapy (ART) in decreasing morbidity and mortality due to HIV cannot be understated. This medication works by preventing the virus from replicating; consistent therapy allows for viral suppression. Importantly, continuous treatment hinders mutated strains from quickly multiplying and leading to drug resistance. Prevention strategies include the advent of pre-exposure prophylaxis (PrEP), which was first approved by the US Food and Drug Administration (FDA) in 2012 [3]. 

Unfortunately, however, many disparities in HIV prevention, incidence, and progression to AIDS still exist. For example, barriers to access and implementation of PrEP remain prevalent in several high-risk populations, including minority groups and those who lack health insurance [4]. Additionally, many difficulties persist in maintaining HIV care and medication adherence [5]. 

In particular, there is a known relationship between decreased socioeconomic status (e.g., lower income or education level) and decreased likelihood of viral suppression. A multitude of other factors, including but not limited to transportation problems, housing or food insecurity, decreased health literacy, and untreated mental health conditions, contribute to ART treatment interruptions and subsequent difficulty in achieving an undetectable HIV viral load [6]. While this article primarily focuses on those living in the United States, these issues are compounded in countries that may not have routine, accessible testing and treatment supplies, leading to dramatically differing retention rates and outcomes. 

It also must be acknowledged that healthcare systems themselves often perpetuate these already existing challenges. For example, people may wait a long time to secure an appointment, which ultimately may be scheduled at a time when they have work or at a location to which transportation is onerous; providers may not communicate in a way that helps patients feel involved and invested in their diagnosis and treatment plans; once being prescribed medication, patients may experience insurance or financial issues; or care among specialists (infectious diseases, primary care, mental health, social work) may not be integrated and coordinated. Treatment interruption due to factors beyond patients' control can lead to viral replication and immune system damage, which in turn opens the door for opportunistic infections. Effective implementation of solutions to personal and systemic barriers in care is being explored actively, both in general and in special patient populations such as pregnant women, youth, and those with unmet social needs [7-9]. 

This case report describes the clinical course of HIV infection in a young person facing multiple barriers to continuous HIV care. As described in this article, lack of treatment for people with HIV continues to be potentially devastating. The aim of this work is to call attention to the consequences of ongoing, systemic barriers to HIV treatment as well as to emphasize that strategies for lowering barriers to HIV treatment continue to warrant aggressive investigation despite the progress seen in recent years. 

This work was presented as a poster at the 2025 Tri-Service Chapters American College of Physicians Annual Scientific Meeting in Virginia Beach in September 2025. 

## Case presentation

At 16 years of age, this female patient was initially diagnosed with HIV (viral load 38,300 copies/mL, CD4 count 236 cells/mm^3^) through routine testing during her first pregnancy (Table 1). She began ART but was subsequently lost to follow-up after moving to another state. The patient was unable to obtain her prescribed medications during this time. Upon relocating to her hometown and re-establishing care during her second pregnancy at age 20, her viral load exceeded 15,000 copies/mL with a CD4 count of 222 cells/mm^3^, and ART was re-initiated (dolutegravir plus tenofovir plus emtricitabine) (Table 1). Throughout this pregnancy, she experienced several interruptions in treatment. At age 26, she continued to lack consistent HIV treatment and was experiencing housing insecurity. While hospitalized for suspected superior mesenteric artery syndrome and new-onset heart failure, her viral load had risen to 48,300 copies/mL, while her CD4 count was less than 20 cells/mm^3^ (Table 1). 

**Table 1:** Patient's abnormal HIV labs (including HIV viral load, CD4 absolute count, and CD4%) across multiple encounters This patient presented during her first pregnancy at age 16 with abnormal HIV labs, including a high HIV viral load, low CD4 count, and low CD4%. A normal HIV viral load is considered "not detected", while a viral load of over 1,000 copies/mL is unsuppressed and >100,000 is very high. A CD4 count above 490 cells/mm^3^ and a CD4% above 30% are normal, while a CD4 count below 200 cells/mm^3^ and a CD4% less than 14% are diagnostic of AIDS. Though initiated on ART at this time, she continued to lack consistent treatment. When she re-presented during her second pregnancy at age 20, her CD4 count and CD4% had both decreased. She was repeatedly lost to follow-up. By age 26, her viral load had continued to rise while her CD4 count and CD4% had decreased, and she was officially diagnosed with AIDS. During her last hospital stay, shortly before being discharged to hospice, her viral load exceeded 3.7 million copies/mL while her CD4 count dropped to a mere 11 cells/mm^3^. The patient never achieved an undetectable HIV viral load nor a normal CD4 count. Reference ranges were derived from the authors' institution. HIV: human immunodeficiency virus; ART: antiretroviral therapy; AIDS: acquired immunodeficiency syndrome; /mL: per milliliter of blood; /mm^3^: per cubic millimeter of blood

	Patient value (age 16)	Patient value (age 20)	Patient value (age 26)	Patient value (age 27)	Reference range
HIV viral load (copies/mL)	38,300	15,700	48,300	3,720,000	Not detected: normal; >1,000: unsuppressed; >100,000: very high
CD4 absolute count (cells/mm^3^)	236	222	<20	11	490-1,740: normal; <200: AIDS (severely immunocompromised)
CD4%	21.0	17.0	7.0	10.6	30.0-61.0: normal; <14.0: AIDS (severely immunocompromised)

The patient was officially diagnosed with AIDS. She was discharged on trimethoprim/sulfamethoxazole (TMP/SMX) (for prophylaxis against *Pneumocystis jirovecii *pneumonia (PJP) and *Toxoplasma gondii *encephalitis) as well as azithromycin (for prophylaxis against *Mycobacterium avium *complex (MAC) disease). Patients with low CD4 counts are more susceptible to these fungal (PJP, CD4 below 200 cells/mm^3^), parasitic (*Toxoplasma gondii*, CD4 below 100 cells/mm^3^), and bacterial (MAC, CD4 below 50 cells/mm^3^) opportunistic infections. 

**Figure 1:**
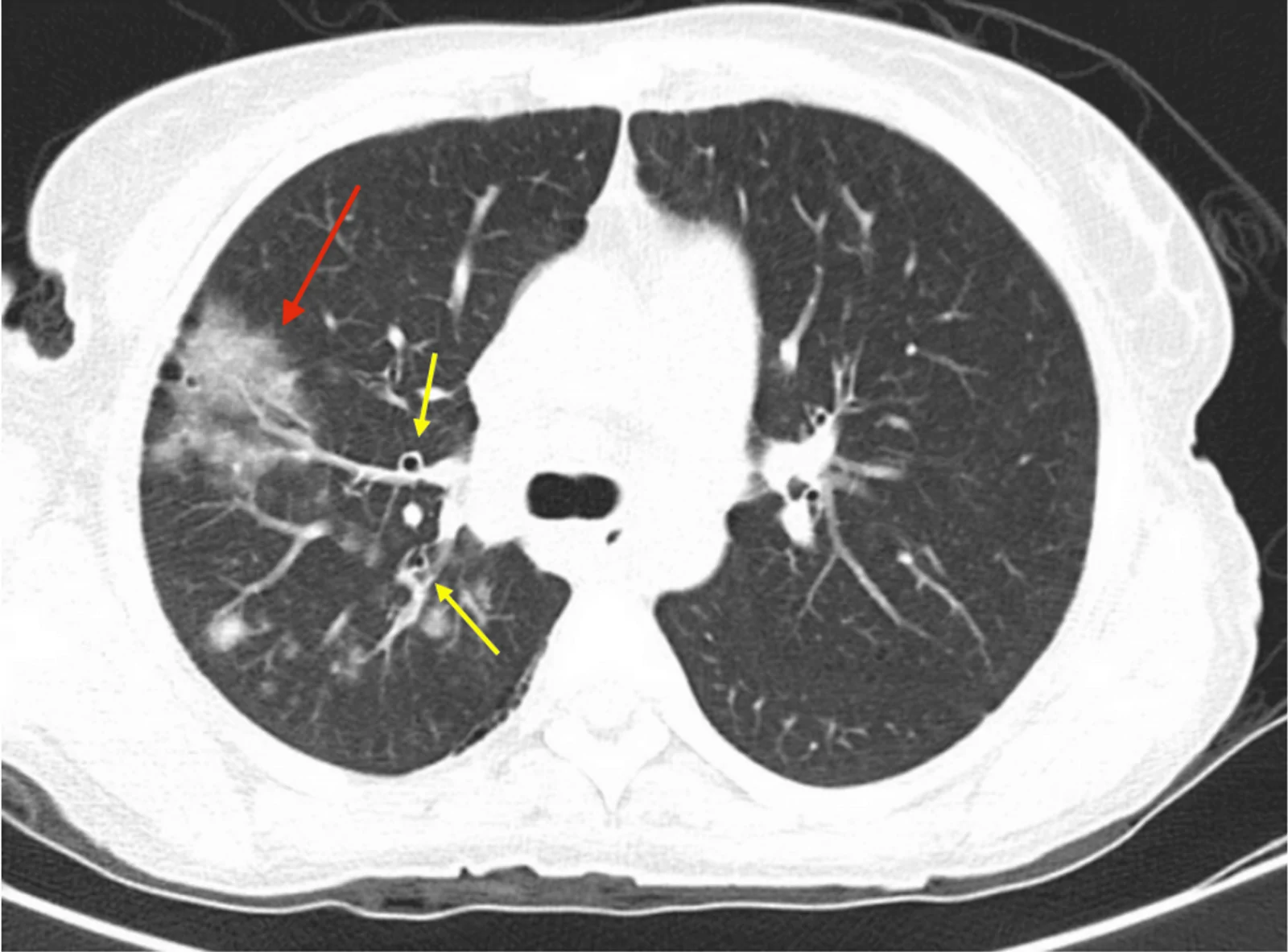
Chest non-contrast CT scan demonstrating ground-glass opacities (red arrow) and multiple cavitary components (yellow arrows) in the right lung consistent with pneumonia During one of her many hospital admissions after being diagnosed with AIDS, the patient was found to have pneumonia. Her imaging, which demonstrates ground-glass opacities (red arrow) and cavitary components (yellow arrows), can be seen above. The diagnosis of pulmonary cryptococcosis was also confirmed on laboratory testing. Pneumonia is common in people with severely suppressed immune systems due to the body's inability to fight off lung infections. This illness was only one of the many sequelae this patient faced as a result of barriers to continuous HIV treatment. CT: computed tomography; AIDS: acquired immunodeficiency syndrome; HIV: human immunodeficiency virus

However, the patient was unable to obtain her medications due to a variety of factors (including being unable to afford them) and subsequently was re-admitted to the hospital a few months later with cough, fevers, headache, nausea, and vomiting. She was diagnosed with acute renal failure, pulmonary cryptococcosis causing pneumonia (confirmed with lung cultures) (Figure 1), cryptococcal meningitis (confirmed with cerebrospinal fluid (CSF) cultures), and sepsis due to *Cryptococcus *fungemia (confirmed with positive blood cultures and antigen), for which she began a course of amphotericin B and fluconazole. Notably, on analysis of labs compared to her previous hospital stay, her kidney function had declined while her liver enzymes had risen. Her month-long hospitalization additionally revealed depression. After discharge, she was lost to follow-up. 

She presented two months later with altered mentation and sepsis; her hospitalization lasted over two months. Upon arrival, she was diagnosed with disseminated MAC (confirmed with positive blood cultures and positive acid-fast bacillus bone marrow biopsy), cryptococcal meningitis, and worsening renal failure. She was re-initiated on ART (dolutegravir plus tenofovir plus emtricitabine). Although she declined a confirmatory kidney biopsy, it was thought that her renal failure was likely secondary to HIV-associated nephropathy (HIVAN) complicated by distal renal tubular acidosis due to amphotericin treatment. Her nephrology team suspected HIVAN due to a nearly 5.5-point increase in her creatinine level from a year prior in conjunction with severe nephrotic-range proteinuria.

At that time, her PJP prophylaxis was changed from TMP/SMX to atovaquone, and her kidney function slowly improved. She also was found to be profoundly thrombocytopenic and anemic with epistaxis, vaginal bleeding, rectal bleeding, and melena, which gradually improved with the re-initiation of ART.

During her hospitalization, she was treated for several opportunistic infections, including disseminated MAC (treated with azithromycin 500 mg daily, ethambutol 1000 mg three times per week, and rifabutin 150 mg every other day), cryptococcal meningitis (induction with liposomal amphotericin followed by renally dosed fluconazole 400 mg every day), herpes simplex virus (treated with acyclovir 550 mg followed by prophylactic dosing of 200 mg twice a day), and trichomoniasis (treated with metronidazole 500 mg twice a day). She was also treated for *Clostridioides difficile *with vancomycin. Overall, her increasing number of opportunistic infections and complications with each inpatient hospital stay were closely correlated with her declining immunologic function as represented by her decreasing CD4 count and CD4% (Table 1). 

While inpatient, she often declined labs, medications, and therapy sessions, contributing to suboptimal nutritional status and functional decline. She was deemed to have capacity and left the hospital against medical advice. She was discharged with medications for MAC and *Cryptococcus.* However, the hospital pharmacy was unable to provide her ART prior to her leaving the hospital. She was scheduled to follow up with an infectious disease physician, but she missed the appointment.

Later that year, the patient was readmitted for stage 4 chronic kidney disease in addition to numerous ongoing complications of her previously diagnosed conditions (including severe malnutrition, debility, anemia, thrombocytosis, heart failure, and several electrolyte abnormalities). Given that she had been diagnosed with several severe illnesses at this point along with extremely deteriorated ability to complete activities of daily living and a consistent wish to turn down almost all medical treatments, palliative care was consulted. After extensive counseling with her providers, including palliative care, she agreed to hospice at the young age of 27.

## Discussion

After several years of treatment interruptions, this patient's health progressed to advanced disease and severe immunocompromise. While ART was initiated multiple times, she was unfortunately not successfully retained in care. She faced numerous socioeconomic challenges, including housing and food insecurity, exacerbated by the healthcare system's inability to mitigate her barriers to continuous treatment. She was also diagnosed with depression yet never secured consistent treatment for her condition; the potential link between her mental health and active refusals of care while hospitalized suggests that treatment resistance is complex and multifactorial. This patient was ultimately failed by the healthcare system across multiple levels, including discharge planning, medication delivery, housing support, and social services coordination. Patients should be afforded a holistic and multidisciplinary approach to healthcare, including not only specialists in infectious diseases but also primary care, mental health, and social services.

Unfortunately, the healthcare system did not adequately mitigate this patient's barriers to care and instead likely unintentionally exacerbated them. Consequently, at a young age, this patient was diagnosed with multiple serious illnesses that impacted nearly all major organ systems. She experienced profound complications involving the immune, neurologic, cardiovascular, pulmonary, gastrointestinal, renal, and hematologic systems. In addition, her immunocompromised state necessitated repeated hospitalizations and treatment for opportunistic infections, including *Cryptococcus *and MAC. Her medical case was especially challenging due to the coexistence of multiple infections and advanced disease affecting numerous organ systems.

The physical effects of medications typically used to manage this patient's condition can have deleterious side effects and increasingly required her providers to weigh the risks and benefits of treatment. It is important that providers remain aware of the potential side effects that may result from treatment in patients with complex health conditions. A high level of suspicion must be maintained when considering the management of patients who present with deteriorating performance status. Careful attention is particularly warranted in those who are already at high risk of poor outcomes.

It is widely recognized that numerous challenges to HIV therapy adherence exist. For example, financial stress, housing and food insecurity, low health literacy, mental health conditions, and other socioeconomic factors are all potential barriers to continuous treatment [6,9,10]. Similarly, the healthcare system's failure to prevent multiple lapses in treatment led to this young woman's progressive and severe decline. Particularly concerning is that many of the health conditions she suffered may have been prevented with appropriate, structured interventions to manage her HIV. The patient did show some level of willingness to participate in treatment during her hospital stays and showed improvement in her symptoms on the multiple occasions that ART was re-initiated; however, she invariably was unable to maintain her treatment regimen, indicating a broader failure of the healthcare system.

This patient's trajectory illustrates how systemic failures in care delivery compound individual barriers to adherence. Her housing insecurity, inability to obtain prescribed medications after discharge, and repeated loss to follow-up reflect not only personal challenges but also inadequacies in healthcare system design. Her unfortunate outcome highlights the need for further investigation on how to effectively and consistently deliver treatment to patients at high risk for progressive disease, especially those with complicating socioeconomic factors.

Overall, a more coordinated and comprehensive approach to care may have altered this young woman's course. For example, while some interventions were attempted (such as repeated counseling, prophylaxis, treatment initiation, and scheduled calls to confirm physician visits), she was not adequately assisted in obtaining medications in a timely manner after discharge and in successfully transitioning from inpatient to outpatient treatment. Reliance on patients to navigate these complexities on their own represents a fundamental flaw in healthcare and, as demonstrated by this case report, predictably results in treatment gaps for vulnerable populations.

Indeed, it is the responsibility of the healthcare system at large not only to prescribe medications and schedule appointments but also to implement strategies to mitigate potential barriers to treatment. Strategies may include an individualized, proactive root-cause analysis examining and subsequently addressing barriers to care, such as (for this patient) housing instability compounded by inconsistent access to communication with providers and lack of transportation; for example, perhaps some of this patient's challenges could have been overcome with the help of mobile healthcare units or accessible virtual health visits. There are several other interventions that have shown promise for increased medication adherence, such as enhanced standard of care and device reminders [7,8], strategies related to cognitive behavioral therapy and self-monitoring [11], and mobile health technology and region-based interventions such as stigma-reduction campaigns in the South [12-15], where HIV prevalence and HIV-related disparities remain high.

Additionally, clinic-level barriers including inflexible appointment schedules, complex documentation requirements for subsidized care programs, poorly resourced hospital-to-clinic transitions, and inadequate systems to identify patients at risk of disengagement must be addressed [16-19] along with systemic failures that place the burden on patients to navigate complex insurance systems, pharmacy access, and social service referrals on their own.

Ultimately, addressing barriers to treatment adherence requires recognition that adherence is not solely an individual responsibility, as there are complex relationships at play on patient, clinic, and systemic levels [16,17]. Healthcare systems must implement structural interventions to overcome these barriers. Evidence-based approaches include the following: (1) multidisciplinary care teams incorporating case managers, social workers, and patient navigators who can address the social determinants of health; (2) direct access to essential services such as housing assistance, nutrition programs, and transportation support; (3) flexible clinic scheduling and community-based care options that reduce navigation complexity; (4) streamlined processes for medication access through AIDS Drug Assistance Programs and pharmaceutical assistance programs; and (5) care delivery models that tailor the location, intensity, and frequency of services to individual needs [16-19].

Modern HIV prevention and treatment medications are extremely effective. However, many barriers to continuous treatment still exist. As this report emphasizes, it is vital to rigorously implement strategies to increase long-term adherence, especially for high-risk populations. The consequences of barriers to continuous care can be catastrophic, such as for the young woman whose case is presented here.

## Conclusions

Despite many established and emerging pharmacologic options for ART in people with HIV, there are ongoing challenges in managing barriers to treatment. These obstacles present a serious dilemma in preventing treatment interruptions and progression to AIDS. Unfortunately, those who develop AIDS are susceptible to numerous opportunistic, concomitant infections and associated comorbidities that can lead to serious and potentially deadly outcomes. In this case, the patient unfortunately went on to require hospice services at only 27 years of age. The healthcare system did not respond adequately to the multifactorial challenges she faced and did not ensure structured support of treatment continuity. 

Therefore, comprehensive strategies addressing both individual and systemic barriers warrant continued implementation and investigation. Patient-centered approaches must be embedded within healthcare systems that actively reduce structural obstacles to care. Specific evidence-based interventions include multidisciplinary care coordination with dedicated case management and social work support; direct access to housing, food, and transportation assistance; streamlined medication access through assistance programs; flexible scheduling and community-based care delivery; and individualized treatment plans. Successful care requires not only effective medications but also healthcare systems that are structurally designed to support the most vulnerable populations in accessing and maintaining treatment. 
